# Secular trend in candidemia and the use of fluconazole in Finland, 2004-2007

**DOI:** 10.1186/1471-2334-10-312

**Published:** 2010-10-28

**Authors:** Eira Poikonen, Outi Lyytikäinen, Veli-Jukka Anttila, Irma Koivula, Jukka Lumio, Pirkko Kotilainen, Hannu Syrjälä, Petri Ruutu

**Affiliations:** 1 Department of Medicine, Peijas Hospital, Vantaa, Helsinki, Finland; 2 Department of Infectious Disease Surveillance and Control, National Institute for Health and Welfare, Helsinki, Finland; 3 Department of Medicine, Helsinki University Central Hospital, Helsinki, Finland; 4 Department of Medicine, Kuopio University Hospital, Kuopio, Finland; 5 Department of Internal Medicine, Tampere University Hospital, Tampere, Finland; 6 Department of Medicine, Turku University Central Hospital, Turku, Finland; 7 Department of Infection Control, Oulu University Hospital, Oulu, Finland

## Abstract

**Background:**

In a previous study we observed an increasing trend in candidemia in Finland in the 1990s. Our aim was now to investigate further population-based secular trends, as well as outcome, and evaluate the association of fluconazole consumption and prophylaxis policy with the observed findings.

**Methods:**

We analyzed laboratory-based surveillance data on candidemia from the National Infectious Diseases Register during 2004-2007 in Finland. Data on fluconazole consumption, expressed as defined daily doses, DDDs, was obtained from the National Agency for Medicines, and regional prophylaxis policies were assessed by a telephone survey.

**Results:**

A total of 603 candidemia cases were identified. The average annual incidence rate was 2.86 cases per 100,000 population (range by year, 2.59-3.09; range by region, 2.37-3.85). The highest incidence was detected in males aged >65 years (12.23 per 100,000 population). *Candida albicans *accounted for 67% of cases, and *C. glabrata *ranked the second (19%), both without any significant change in proportions. *C. parapsilosis *accounted for 5% of cases and *C. krusei *3% of cases. The one-month case-fatality varied between 28-32% during the study period. Fluconazole consumption increased from 19.57 DDDs per 100,000 population in 2000 to 25.09 in 2007. Systematic fluconazole prophylaxis was implemented for premature neonates, patients with acute leukemias and liver transplant patients.

**Conclusion:**

The dominant proportion of *C. albicans *remained stable, but *C. glabrata *was the most frequent non-*albicans *species. The proportion of *C. glabrata *had increased from our previous study period in the presence of increasing use of fluconazole. The rate of candidemia in Finland is still low but mortality high like in other countries.

## Background

Bloodstream infections caused by *Candid*a spp. represent a considerable burden in hospitalized patients and are associated with high mortality and an excess length of stay [1-5]. *Candid*a is an important cause of bloodstream infections worldwide. In the USA *Candida *species rank the fourth most common (7-9%) causative organism of all bloodstream infections in hospitalized patients,[6-8] but in Europe it accounts for less than 5%[9-11].

Despite the considerable burden from candidemia in hospitalized patients, most population-based studies are from the USA [12-15]. The reported population-based incidences of candidemia have increased over the past two decades, though this varies between countries and the time periods in many analyses are relatively short [12-19]. Population-based analyses avoid the selection bias associated with single institutions or tertiary care centers allowing calculations of incidence and mortality rates. The reasons for the increasing candidemia rates include improved detection as well as increase in patient-population at risk, as invasive procedures and devices, broad-spectrum antimicrobial agents, advanced life-support and aggressive chemotherapy are more extensively used.

A shift towards non-*albicans *species has been reported previously from the USA, Europe and Australia, although the precise pattern of causative species varies across countries [13,14,16,20-27]. The observed increase in proportion of non-*albicans *species, especially *C. glabrata*,[10,12,16,20,22,23,26,28] has aroused concern due to its tendency toward decreased susceptibility to fluconazole.

Previously, we reported a low, but increasing incidence of candidemia during 1995-1999 in Finland [29]. Our present study reports the trends in incidence rates of candidemia in Finland from 2004 to 2007, using data on bloodstream infections from laboratory-based nationwide surveillance. We also analyzed fluconazole consumption during 2000-2007, and assessed the policy of fluconazole prophylaxis in all tertiary care centers in Finland.

## Methods

### Surveillance

In Finland (population 5.3 million), the national health care system is organized into 5 geographically and administratively defined tertiary care districts, with populations ranging from 0.72-1.76 million.

Since 1995, all clinical microbiology laboratories in Finland have notified all bacterial and fungal isolations from blood, including *Candida *species, to the National Infectious Diseases Register maintained by the National Institute of Health and Welfare. Detection and species identification of *Candida *isolates are performed in the notifying laboratories according to standard protocols in use in each laboratory. Data collected with each notification include the date of isolation, the date of birth, sex, the type of specimen, and the place of treatment. A case of candidemia was defined as a patient with at least one blood culture positive for *Candida *species. Notifications of the same species of *Candida *within 3 months from the first diagnostic sample in the same person were defined as one case. Isolation of the same species beyond this time period was defined as a separate case.

### Consumption of fluconazole

Data on fluconazole consumption in defined daily doses, DDDs, during 2000-2007 was obtained from the National Agency for Medicines.

### Survey on prophylactic policy

We interviewed infectious diseases specialists in the five tertiary care hospitals per telephone using a structured questionnaire about written guidelines or routine practice for fluconazole prophylaxis in their hospital among following patient groups, adults (≥15 years) and children (<15 years) separately: patients with hematologic or other malignancy, patients treated in intensive care, surgical patients, organ transplant patients and premature neonates.

### Calculation of incidence rates and statistical analysis

Using the patients' unique national identity number for register linkage, individual death data were acquired for the period 2004-2007 from the National Population Information System for determining survival at 28 days from the first positive blood culture. Denominators to calculate age- and sex-specific incidence rates were acquired from the same register. The average annual incidences during the surveillance period were calculated by using the total number of cases and population during 2004-2007. To evaluate secular trends, rates of candidemia cases in different age and sex groups were calculated for each 12-month period from January 2004 to December 2007. Poisson regression model was used to assess whether the observed changes in the rates or fluconazole consumption were statistically significant. Categorical variables were analyzed with the χ^2 ^test, using Yates`s correction, or Fisher`s exact test, as appropriate. Continuous variables were analyzed by Student`s *t *test or by the Mann-Whitney *U *test, depending on the sample distribution. Data were analyzed by SPSS for Windows version 11 (Chicago, USA).

### Data approval

The data of National Infectious Diseases Register is publicly available in annual reports of National Institute of Health and Welfare, although not as detailed form as in our study. The data of fluconazole consumption is publicly available in annual reports of National Agency for Medicines. The detailed analysis prepared in our study was authorized by these both authorities holding the original data. The data for calculations from the National Population Information System for years 2004-2007 was authorized by National Institute of Health and Welfare in connection with National Infectious Diseases Register.

## Results

### Demographics and incidence

A total of 603 candidemia cases were reported to NIDR during 2004-2007. The median age of the case-patients was 64 years (range, 0-94 years,) and 337 (56%) were males. The average annual incidence of candidemia was 2.86 cases per 100,000 population (range by year, 2.59-3.09 per 100,000) without any trend during the study period.

The average annual incidence was significantly higher in males than in females, especially among patients >65 years of age (Table 1). The highest average annual incidence was observed in males >65 years of age, and lowest in males 1-15 years of age. Only in males 16-65 years of age the annual rate slightly increased during the study period, from 2.11 to 2.65 per 100,000 population, but the trend was not statistically significant.

**Table 1:** Incidence of candidemia by age and sex, Finland 2004-2007.

Age group (y)	Male		Female		All		Rate ratio**	Confidence interval (95%)
	**Number of cases**	**Rate**	**Number of cases**	**Rate**	**Number of cases**	Rate*		

<1	11	9.24	5	4.39	16	6.87	2.1	0.36-4.13
1-15	1	0.05	8	0.45	9	0.25	0.12	0.03-0.89
16-65	169	2.39	126	1.82	295	2.11	1.31	1.04-1.67
>65	156	12.23	127	6.57	283	8.82	1.86	1.46-2.37
All	337	3.27	266	2.47	603	2.86	1.32	1.12-1.56

*Average incidence during the study period (per 100,000 population)

**Male to female rate ratio

The average annual incidence rates varied between 2.37-3.85 per 100,000 population in the five tertiary care districts; the incidence increased in three districts, but the trends were not statistically significant (Table 2). The highest annual incidence rate was observed in Oulu in 2006, and the lowest in Helsinki in 2005.

**Table 2:** Candidemia incidence and proportion of non-albicans species by tertiary care center districts, Finland 2004-2007.

	Incidence of candidemia, cases per 100,000 population				
	(Proportion of non-albicans species, %)				
	
Region (population)	2004	2005	2006	2007	2004-2007
Helsinki (1.76 million)	2.92 (41)	1.82 (22)	2.32 (24)	2.69 (33)	2.44 (31)
Tampere (1.2 million)	3.00 (25)	2.74 (42)	3.64 (21)	3.45 (36)	3.21 (30)
Turku (0.72 million)	2.24 (44)	2.66 (42)	3.07 (27)	3.63 (35)	2.9 (36)
Oulu (0.73 million)	3.31 (30)	3.58 (38)	4.54 (36)	3.98 (18)	3.85 (30)
Kuopio (0.85 million)	2.34 (20)	3.05 (27)	1.88 (31)	2.23 (26)	2.37 (26)
All 5 regions (5.2 million)	2.81 (33)	2.59 (34)	2.96 (27)	3.09 (30)	2.86 (31)

### Causative species

**Figure 1:**
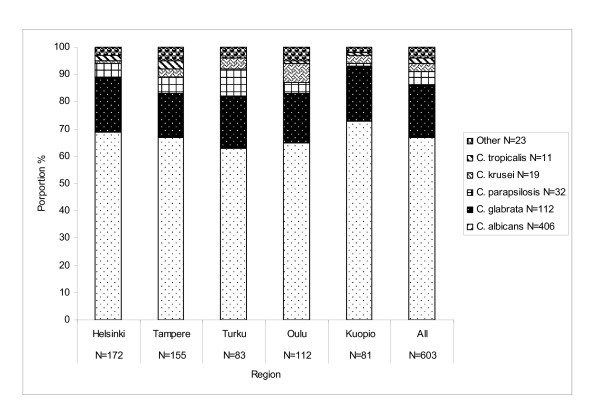
**Distribution of Candida species by region (tertiary-care center catchment area), Finland 2004-2007**.

*Candida albicans *was the most frequent species encountered, causing 406 (67%) cases and *C. glabrata *ranked second (19%), followed by *C. parapsilosis *(5%), *C. krusei *(3%), and *C. tropicalis *(2%) (Figure 1). The other species reported were *C. lusitaniae*, *C. guilliermondii *and *C. dubliniensi*s; two species were reported in 7 (1%) cases, and in 4 (1%) cases the *Candida *species was unknown. During the study period, the proportions of *C. glabrata *and non-*albican*s species overall did not increase. Among the five tertiary care districts, the proportion of non-*albicans *species peaked during 2004-2005 in Turku at 44-42% and 2004 in Tampere at 42%, mainly consisting of *C. glabrata *and *C. parapsilosis *(Table 2). Thereafter, the proportion of non-*albicans *species remained lower (range, 18-36%) in all districts.

### Mortality

Data on outcome was available for 598 (99%) case-patients, 208 (35%; range by year, 32-38%, and range by district, 31-37%) of whom died within one month after the onset of candidemia. The average annual mortality rate was 0.99 deaths per 100,000 population (range by year, 0.89-1.08, and range by district, 0.91-1.27). The case-patients who died were significantly older than those who survived (median age, 70 *vs *62 years; P < 0.01).

### Fluconazole consumption

**Figure 2:**
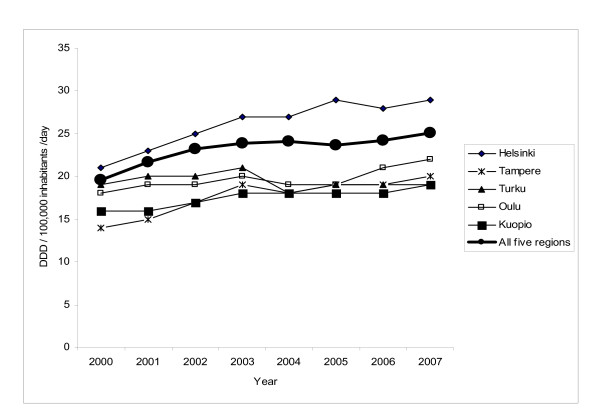
**Use of fluconazole in daily defined doses (DDD) by region (tertiary-care center catchment area) in Finland, 2000-2007**.

Fluconazole consumption increased significantly from 19.57 DDDs per 100,000 population in 2000 to 25.09 in 2007 (P < 0.01) (Figure 2). The increase in consumption was statistically significant in all five regions (P < 0.01). Per oral consumption covered 89% (range by year, 88-90%) of the total fluconazole use, the rest was parental use.

### Prophylactic policies

Fluconazole prophylaxis was used in all five tertiary care centers for adult acute leukemia patients treated with intensive chemotherapy, over each period of neutropenia, mostly from 2000 on and in one center since 2007. Written guidelines were in place in three of these centers. Pediatric patients with acute myeloid leukemia were on fluconazole prophylaxis during neutropenia caused by intensive chemotherapy in four out of the five centers since 1998-2000 on (written guidelines existed in three); patients with acute lymphatic leukemia were administered fluconazole prophylaxis during neutropenia in two centers (written guideline existed in one).

Fluconazole prophylaxis was administered for adult stem cell recipients in one center out of five according to a written guideline since 2007. Pediatric autologic stem cell recipients were on fluconazole prophylaxis in four out the five centers during hospital stay or neutropenia mostly since 2000 on (written guideline existed in two centers).

Allogenic stem cell transplantations for pediatric patients were performed only in three tertiary care centers for all regions and fluconazole prophylaxis was administered according to a written guideline in all three centers for at least during hospital stay and in one center for 6 months since 2000 on. Solid organ transplantations were performed solely in one tertiary care center for all regions. Only liver transplantation patients (adults and children) were on fluconazole prophylaxis starting right before or at the operation and lasting at least 5 days postoperatively from 1998 on, without written guidelines.

Premature infants received fluconazole prophylaxis usually for 2-6 weeks or during invasive monitoring in four out of the five tertiary care centers; written guidelines existed in two. Prophylaxis was used at least since 2002-2004 on in all four centers. No guidelines for fluconazole prophylaxis to adult or pediatric intensive care patients or surgical patients were in place in any tertiary care center.

## Discussion

Our population-based study shows that the incidence of candidemia in Finland was low and stable during 2004-2007 in the face of increased fluconazole consumption. *C. glabrata *ranked second to *C. albicans *in proportion, but the proportion of non-*albicans *species did not increase.

Compared with our previous nationwide population-based data from Finland during 1995-1999, the average annual incidence of candidemia has increased markedly from 1.9[29] to 2.86 per 100,000 population in the current study period. The rate in Finland is only approximately one third of the rates reported from the USA, most reports ranging between 6-10 per 100,000 population, while a considerably higher incidence of 24 per 100,000 population has been reported from the city of Baltimore [12-15]. Low incidence rates of 2.4-2.8 per 100,000 population have also been reported from Norway and Canada,[19,30] while incidences have been somewhat higher elsewhere in Europe,[18,31] and considerable higher (10.4 per 100,000 population) in Denmark [16]. Extended surveillance periods have been previously reported from Iceland for 1980-2006, and from Norway for 1991-2003; the incidences increased from 1.4 to 5.8 and from 2 to 3 per 100,000 population, respectively [17-19]. Thus, the increasing rate in Finland between the periods in the 1990s and the 2000s highlights the growing importance of *Candida *spp. in hospitalized patients. The marked differences in candidemia rates between countries may reflect differences in representativeness and age distributions of the study populations, variations in health care practices, patterns using blood cultures, and antibiotic usage as well as the resistance situation.

The male dominance we observed during 2004-2007 is in accordance with our previous study from years 1995-1999, and with most reports both from USA and Europe,[12,14,16,19,29] but in contrast with some others [13,30]. By age and sex, the incidence rates increased most in patients >65 years of age, from 5.2 during 1995-1999[29] to 8.82 per 100,000 population in the current study, particularly in males, being in the same range as in Norway and Spain,[19,31] but clearly lower than in Denmark, Iceland and USA (19.3-36.9 per 100,000 population) [14,16,17]. Interestingly, in the two youngest age groups (<1 year and 1-15 years of age), the incidence was lower during 2004-2007 than during 1995-1999 in Finland. This is partly explained by fading of an outbreak of *C. parapsilosis *in the neonatal intensive care unit of Helsinki University Hospital from 2002 on [32]. Incidence rates in infants <1 year of age have been clearly higher, 20-75 per 100,000 population, in Northern America and Spain,[14,30,31] and somewhat higher in other Nordic countries, 10.3-16.3 per 100,000 population, than in Finland [16,17,19].

We did not observe any changes in the proportion of *C. albicans *between 1990s[29] and 2000s (current study) in Finland, but the proportion of *C. glabrata *increased considerably from 9% to 19%. Other *Candida *species were isolated only rarely, and between the study periods, the proportion of *C. krusei *decreased from 8% to 3%. The reason for this decrease is elusive, and is not explained by sheer antibiotic pressure, but may associate with patient demographics or underlying conditions. The stable predominance of *C. albicans *we have observed is in contrast to several reports from the USA and Europe, in which the proportion of non-*albicans *species has increased up to 40-50% [10,12-14,18,20,22-27,33]. However, no shift to non-*albicans *species was detected in Norway, Iceland, Belgium and Switzerland [17,19,34-36]. The increase in *C. glabrata *has previously been reported from the USA,[12,20,22-24,26] and from Europe [10,16,28]. The reasons for the increasing proportion of *C. glabrata *may include exposure to azoles or antibacterial agents, differences in patient age, comorbidities and in blood culture systems to isolate *Candida *spp.

The total consumption of fluconazole increased in all five regions in Finland during 2000-2007, which suggests also increased usage of fluconazole in tertiary care hospitals. Previously, we reported high fluconazole consumption in one tertiary care center in Finland, reaching 135 grams per 10,000 patient-days (corresponding to 6750 DDDs per 100,000 patient days) in 2004,[37] in the same range with consumption of 5013-6807 DDDs per 100,000 patient-days during 1994-2004 in Belgium [34]. From earlier time periods, clearly lower fluconazole consumption was reported from a multicenter study in Switzerland, with 30 grams per 10,000 patient-days (corresponding to 1500 DDDs per 100,000 patient-days) in 2000 [36] and in the USA, with >30 grams per 10,000 patient days in 1993 [38].

Prophylaxis with fluconazole was systematically used during our current study period in patients with acute leukemias, liver transplant patients, and in premature infants. The prophylactic use of fluconazole in neonates and pediatric leukemia patients may have contributed to the observed decreasing rates of candidemia in patients <16 years. Despite the increase in fluconazole consumption during 2000s, we did not observe any shift towards non-*albicans *species as a whole, but among non-*albicans *species the proportion of *C. glabrata *increased during 2004-2007 compared to our previous study [29]. The role of fluconazole consumption in the shift of causative *Candida *species is inconclusive, as several reports have observed the effect[20,23,26], while others have not [19,24,34,36].

The one-month case fatality proportion remained stable at 35% over the study period, and was associated with advanced age, like in our previous study from years 1995-1999 [29]. This is in line with most earlier reports from the USA and Europe,[3,13,25,28] although higher rates 44-45% have been reported from some centers [27,33].

Our study has several limitations. This is an ecologic study in which we used the total consumption data on fluconazole in different tertiary-care catchment regions, in connection with our population-based data of incidence rates, making inferences on individual patients based on average population statistics. With only laboratory-based reporting, it is not possible to differentiate between nosocomial and community-onset infection. Consequently, we could not calculate fluconazole consumption as DDDs pe patient-days. In our previous study from one tertiary care center during 1987-2004 the proportion of non-nosocomial infections was 4% [37], but we do not know what proportion of our present data represents of nosocomial candidemias. We do not have data on sensitivity to fluconazole. Guidelines and routine prophylaxis policies presented are limited to patients in tertiary care centers, and thus affect the candidemia rates in these hospitals, and not on health-care institutions as a whole. However, the infectious diseases physicians of the centers have a key consulting role in their districts on policies regarding high risk groups for candidemia.

## Conclusions

Our study demonstrates that candidemia has increased in Finland between 1990s and 2000s, although the incidence still remains internationally low. Fluconazole prophylaxis in hematological patients and neonates is systematically implemented, while no systematic prophylaxis is used in the intensive care setting. The increasing proportion of *C. glabrata *is of concern, but this increase may be caused by other risk-factors than increasing fluconazole usage. The high case-fatality emphasizes the need for continuing surveillance to optimize prevention policies, including antifungal prophylaxis.

## Competing interests

The authors declare that they have no competing interests.

## Authors' contributions

EP, OL and PR conceived the framework and drafted and finalized the manuscript. V-JA, IK, JL, PK and HS participated in the prophylaxis survey and by gathering the data on local guidelines and recommendations, and also finalizing the manuscript. All authors read and approved the final manuscript.

## Pre-publication history

The pre-publication history for this paper can be accessed here:

http://www.biomedcentral.com/1471-2334/10/312/prepub
